# Quantitative determination of embolization endpoints based on local arterial pressure

**DOI:** 10.1002/btm2.70078

**Published:** 2025-10-16

**Authors:** Dongcheng Ren, Xingyuan Li, Shijie Guo, Yuchi Tian, Baolei Guo, Bo Zhou

**Affiliations:** ^1^ School of Mechanical Engineering Hebei University of Technology Tianjin China; ^2^ Engineering Research Center of the Ministry of Education for Intelligent Rehabilitation Equipment and Detection Technologies Hebei University of Technology Tianjin China; ^3^ Hebei Key Laboratory of Robot Perception and Human–Robot Interaction Hebei University of Technology Tianjin China; ^4^ Institute of Research and Clinical Innovations Neusoft Medical Systems Co., Ltd Shanghai China; ^5^ Department of Vascular Surgery Fudan University Zhongshan Hospital Shanghai China; ^6^ National Clinical Research Center for Interventional Medicine Shanghai China; ^7^ Department of Interventional Radiology Fudan University Zhongshan Hospital Shanghai China

**Keywords:** arterial pressure, embolization endpoint, interventional embolization, porous media, quantitative embolization procedures

## Abstract

This study aims to optimize the embolization endpoint to improve therapeutic outcomes in interventional procedures and minimize the risk of ectopic embolism caused by excessive embolic agent injection. Hemodynamic changes during embolization were simulated by modeling the terminal resistance vessels as a porous medium. An in vitro experimental platform has been developed to replicate the embolization process. Based on these simulations and experimental data, a quantitative method was established to evaluate the embolization endpoint using local arterial blood pressure. The method was further validated through renal artery embolization experiments in pigs. The quantitative method effectively predicted changes in local arterial pressure and flow rate, with an average error of approximately 1.65% in simulations and 3.09% in in vitro experiments. In animal studies, the pressure‐based endpoint evaluation method closely aligned with imaging results, reducing the required embolic agent by an average of 17.86%. Local arterial blood pressure is considered a reliable criterion for determining the embolization endpoint, offering a relatively standardized and quantitative approach to embolization endpoint assessment. This method has significant clinical value in reducing radiation exposure and facilitating the automation of embolic agent injection procedures in the field of embolization therapy for solid tumors.

## Graphical abstract

This study establishes a quantitative embolization endpoint assessment method based on local arterial pressure, integrating CFD simulations and in vitro experiments, enabling accurate, reproducible, and low‐radiation evaluation to optimize embolization safety and preserve normal tissue.

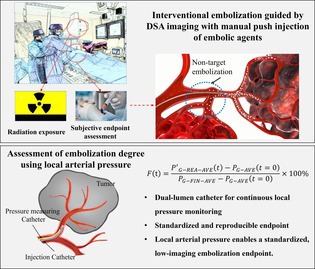

**Translational Impact Statement**

This study presents a pressure‐based method for safer, automated embolization, radiation‐related harm, and improving treatment precision, which could enhance vascular intervention embolization surgery outcomes.

## 1 INTRODUCTION

**FIGURE 1:**
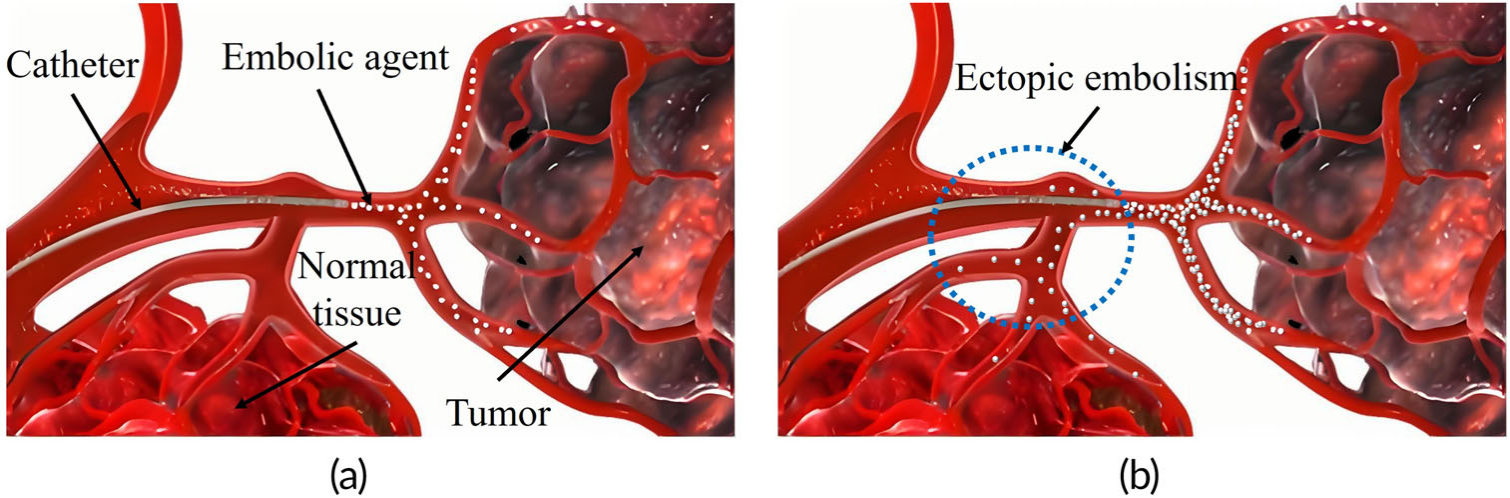
Schematic diagram of ectopic embolism: (a) Normal embolism. (b) Ectopic embolism.

Interventional embolization has revolutionized modern medicine, by significantly improving the treatment of various vascular‐related diseases. Procedures such as uterine fibroid embolization, prostate artery embolization, transcatheter arterial chemoembolization (TACE), and tumor embolization have become standard practices in clinical practices.^1^ The key to successful vascular interventional embolization lies in the precise and controlled delivery of embolic agents to the target vessel. An inadequate dosage of embolic agents may not achieve the desired therapeutic effect, whereas an excessive dosage can obstruct normal blood flow, leading to ectopic embolism (Figure 1) and severe complications.^2^

Imaging techniques are commonly used to determine the endpoint of embolization during clinical procedures.^3^, ^4^ However, reliance on image‐based methods poses significant challenges, including radiation exposure to medical personnel and inter‐observer variability among radiologists, which can lead to inconsistent and subjective endpoint determinations.^5^, ^6^ This variability is further influenced by regional medical practices, settings, and individual radiologists' expertise.^7^

With advancements in robotic technology, the need for automated embolic agent injection systems capable of quantitative and precise control is growing. Achieving a quantitative assessment of embolization status is essential for such robotic systems to assist or even replace human operators in embolic delivery.

Research has demonstrated that direct measurement of blood flow velocity within the target vessel can effectively predict the endpoint of embolization. Periyasamy et al. utilized quantitative digital subtraction angiography (QDSA) to observe changes in hepatic artery blood flow during embolization, although this method increased radiation exposure due to the need for additional imaging.^8^ Borowski et al. noted a significant pressure increase at the distal end of the hepatic artery post‐chemoembolization, but further investigation into the relationship between arterial pressure and endpoint of embolization is necessary.^9^ Gowda et al. introduced a multi‐lumen catheter concept for real‐time pressure measurement upstream of the tumor site during injection, which was validated using an in vitro silicon vascular model. Their study established a correlation between intravascular pressure, blood flow, and injection parameters based on the volume of injected embolic particles. However, their findings were not validated in animal experiments, and a more detailed understanding of the relationship between the embolization endpoint and local pressure is still required.^10^

In our previous works,^11^ we proposed that local arterial blood pressure could serve as a reliable indicator of embolization degree and introduced the concept of “final pressure” as a corresponding endpoint to mitigate the risk of ectopic embolism. This hypothesis has been supported by both vitro and animal experiments.

This paper presents an innovative approach by utilizing “final pressure” as a precise and reliable indicator for determining the endpoint of embolization. Through the employment of a porous medium model to simulate hemodynamic changes and the establishment of an in vitro experimental platform, a novel method was developed to assess endpoint of embolization using local arterial mean pressure. The efficacy of this method was validated through renal artery embolization experiments in pigs.

The main contributions of the study are summarized as follows:

- A novel pressure‐based method for embolization endpoint assessment is proposed, addressing the limitations of current image‐dependent strategies.
- A porous media model is established to simulate hemodynamic changes during embolization, validated through in vitro experiments, thereby contributing to the accurate prediction of embolization outcomes.
- The effectiveness of this method is confirmed through renal artery embolization experiments in pigs, demonstrating its potential for broader clinical application and laying the groundwork for future innovations in automated embolization procedures.

## 2 MATERIALS AND METHODS

### 2.1 Embolization process and unit vascular model

Interventional embolization procedures utilize a variety of embolic agents, including metal coils, liquid or gel‐based substances, and particulate forms. Each type of embolic agent has distinct benefits and limitations, making them suitable for specific clinical applications. Initially, granular embolic agents were developed and used in embolic procedures.^1^ With advancements in microsphere technology, microspheres have become essential materials for embolization in interventional procedures.^12^ Therefore, PVA calibration microspheres were selected for this study to investigate the process of vascular intervention embolization.

A catheter was threaded through the femoral artery into the aorta and, guided by a guidewire, was advanced into the organ arteries. To preserve healthy tissues and ensure precise targeting of diseased areas, the catheter is typically super‐selective, reaching the secondary or even tertiary branches of the organ arteries.^12^

**FIGURE 2:**
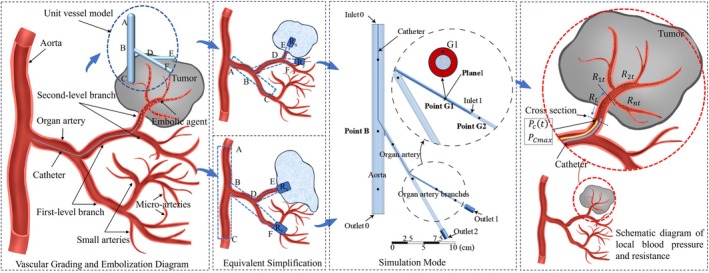
Equivalent modeling and simulation model of blood vessel.

Embolic agent particles, typically ranging from 100 to 700 μm, are injected via microcatheters with inner diameters between 0.7 and 1.3 mm (e.g., microcatheter or 4Fr catheter).^13^ These agents are primarily designed to occlude small arterioles (0.3–1 mm) and microarterioles (<300 μm) at the distal end of the catheter.^14^ According to Poiseuille's law,^15^ the accumulation of embolic particles increases the distal resistance of the vascular bed. To avoid the complexity of explicitly modeling the terminal microvasculature, we employed an equivalent porous media model to represent the embolized region,^16^ as schematically illustrated in Figure 2.

In Figure 2, the vessel where the catheter tip is located is marked as DE, while other vessels originating from the same point are designated as DF. Resistance sources (*R*_*x*_ and *R*_*y*_) are present at the ends of DE and DF, representing the flow resistance at their respective terminations. BD is the superior vessel for both DE and DF, with ABC being the superior vessel of BD. These vessels form a unit vessel model, representing a nested structure of multiple vascular units from the aorta to the target vessel. The blue solid and dashed lines within the vessels depict the simplified state of the vessels at various positions relative to the catheter tip.

During embolization, the total downstream resistance from the pressure‐sensing catheter tip to the venous outlet is denoted as $R\left(t\right)$. It comprises the resistance of the main target artery $R_{L}$ and the cumulative resistance of the distal microvasculature $R_{1t},R_{2t},\ldots R_{\mathrm{nt}}$, which progressively increase as embolic agents accumulate.

To capture the hemodynamic consequences of embolization, we define a simplified pressure–flow relationship at the measurement site as:

(1) $$Q\left(t\right)=\frac{\left(P_{c}\left(t\right)-P\right)}{R\left(t\right)},$$

where $Q\left(t\right)$ represents the residual blood flow perfusing the downstream vasculature at time 𝑡, excluding the embolic agent flow. $P_{c}\left(t\right)$ is the real‐time local arterial pressure recorded at the tip of the pressure‐sensing catheter, and 𝑃 is the terminal venous pressure (assumed to be 0 mmHg for simplicity).

As embolization progresses and distal resistance increases, $P_{c}\left(t\right)$ gradually rises. Based on our previous findings,^11^ this pressure eventually approaches a plateau—the physiological upper limit $P_{\text{cmax}}$. Under this condition, the maximum possible perfusion flow supported by the vascular segment represented by $R\left(t\right)$ is given by:

(2) $$\begin{array}{c} Q_{\max}\left(t\right)=\left(P_{\text{cmax}}-P\right)/R\left(t\right) \end{array}.$$

This quantity reflects the maximum total flow capacity of the downstream vasculature at time *t*, under full perfusion pressure. Accordingly, the theoretical maximum allowable embolic agent flow rate at time *t* is defined as the difference between the maximum capacity and the current residual blood flow:

(3) $$\begin{array}{c} Q_{E\max}\left(t\right)=Q_{\max}\left(t\right)-Q\left(t\right)=\frac{\left(P_{\text{cmax}}-P_{c}\left(t\right)\right)}{R\left(t\right)}. \end{array}$$

This formulation enables real‐time estimation of embolic agent delivery capacity, based solely on measurable arterial pressure and inferred vascular resistance. It also emphasizes the inverse relationship between embolic flow potential and embolization progression. To further model the dynamic increase in resistance, we treat the embolized vascular bed as a porous medium, where the embolic material induces momentum loss. The resulting pressure drops across the embolized segment follows the standard Darcy–Forchheimer equation:

(4) $$\begin{array}{c} P_{c}\left(t\right)=∆P=S_{t}∆n=\frac{∆\mathrm{n\mu}}{\alpha }v_{t}+C\frac{∆n}{2}\rho \left|v\right|v_{t} \end{array},$$

where ∆P is the pressure drop, ∆n is its thickness, $S_{t}$ represents the momentum source term, μ is the dynamic viscosity, v is the velocity, ρ is the density, α is the permeability, and C is the coefficient matrix of viscous inertia resistance.

It should be emphasized that Equations ((1), (2), (3), (4)) are not intended for direct clinical application, but instead provide a theoretical basis for the pressure‐derived embolization index (defined later in Equation (6)). Together, they illustrate how progressive embolization leads to increasing distal resistance and measurable changes in proximal arterial pressure.

### 2.2 Simulation

The simulation model shown in Figure 2 corresponds to the unit vessel model, encompassing the aorta, organ artery, and its branches. The porous media domain represents the terminal resistance vessels at the distal end of the organ artery. In the model, the aorta has a diameter of 20 mm, the organ artery 6 mm, and the branches 3 mm, closely approximating real vessel sizes.^17^, ^18^, ^19^, ^20^, ^21^ The hollow section represents the space occupied by the 4Fr catheter within the arterial branches. As shown in Figure 2, the inlet, outlet, and data acquisition points in the model are consistent with those used in the in vitro experiments.

Mesh generation was performed using the commercial software ANSYS Mesh (ANSYS, Inc., Pennsylvania, USA). The Fluent solver was employed for the simulation. To ensure both accuracy and computational efficiency, local mesh refinement was applied. The element size was set to 0.2 mm in the organ artery branches, 0.1 mm along the catheter surface, 2 mm in the aorta, 1 mm in the organ artery trunk, and 0.5 mm in the porous media domain. Curvature‐based refinement and inflation layers (five layers, growth rate 1.2, transition ratio 0.272) were used to enhance accuracy near vessel bifurcations and catheter boundaries. The mesh quality was further optimized using Fluent's built‐in quality improvement tools. Final mesh orthogonal quality ranged from 0.4 to 0.99, with an average of 0.85. The overall mesh contained 6.75 million elements.

**FIGURE 3:**
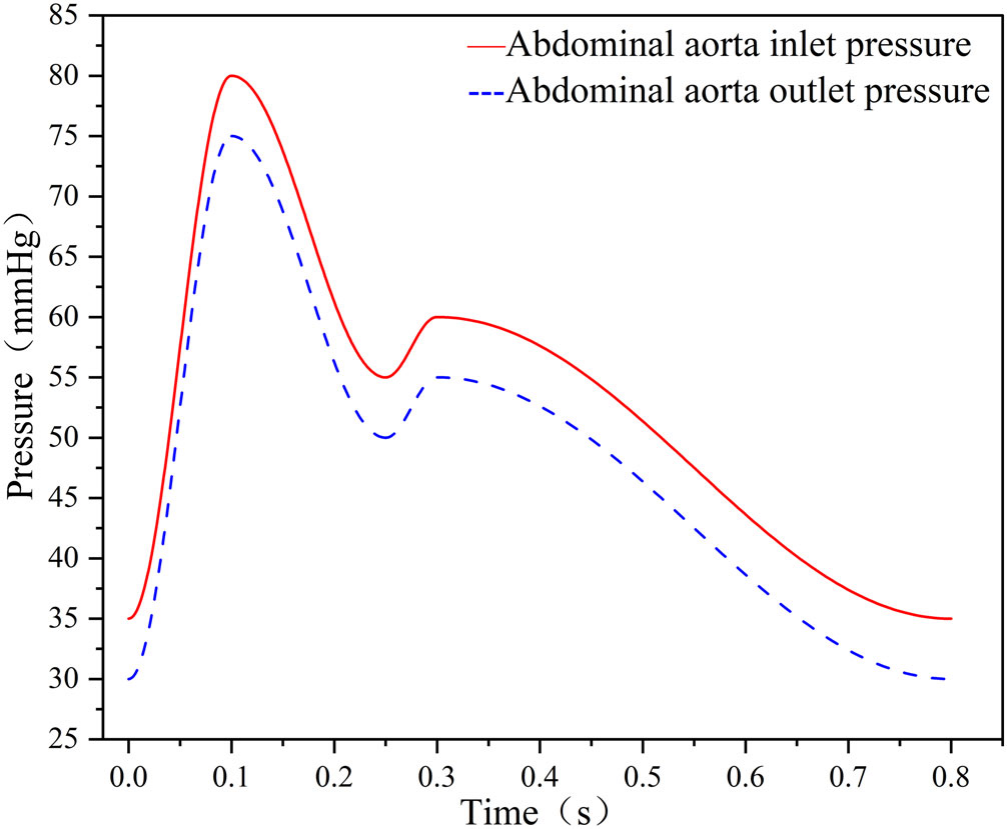
Pressure boundary condition.

Pulsatile flow was simulated using physiologically representative pressure waveforms applied at both the aortic inlet (inlet 0) and the venous outlet (outlet 0), based on the pressure settings used in the in vitro pulse pump (Figure 3). The side branches (outlets 1 and 2) were defined as pressure outlets, while the terminal venous pressure was set to 0 mmHg due to its low amplitude. The flow was modeled as laminar, and blood was treated as a non‐Newtonian fluid using the Carreau model.^22^ The blood density was 1060 kg/m^3^ based on measured animal blood parameters.

To ensure consistency between simulation and experiments, the porous media region was assigned empirical resistance coefficients derived from copper sponge experiments, as detailed in our previous study^11^ and summarized in Table 1. These parameters were fitted using the Darcy–Forchheimer equation over embolization volumes from 0 to 5 mL.

**TABLE 1:** Equivalent resistance coefficients at different embolic doses.

Dose of embolic agent (ml)	Inertial resistance coefficient (10^3^/m)	Viscous resistance coefficient (10^8^/m^2^)
0	9.36	3.19
0.5	10.96	3.25
1	16.18	3.31
1.5	21.78	3.35
2	26.70	6.46
2.5	31.85	10.06
3	37.21	13.81
3.5	41.31	18.74
4	43.75	25.09
4.5	46.36	32.38
5	49.73	37.75

At inlet 1 of the embolic agent, the velocity inlet was set to 0 to simulate the blood flow state when the injection was halted after a certain amount of embolic agent was introduced. Because the injected embolic agent comprises normal saline and contrast agents, its overall density closely resembles that of blood. The simulation aims to observe hemodynamic parameters in the bloodstream rather than the distribution of embolic particles in the blood vessel. The end vessel resistance can be achieved by setting the parameters of porous media. Consequently, in this study, we did not conduct multiphase and particle flow simulations.

In this study, all vessel walls in the simulation model were assumed to be rigid and impermeable. This modeling choice, widely adopted in early‐stage computational hemodynamics studies, allows for stable numerical convergence and significantly reduces computational cost.^23^ While this simplification omits the physiological compliance of arterial walls, it is a practical choice for this study. This assumption is also consistent with the use of rigid phantoms in the in vitro experiments.

Transient simulations were performed across embolization stages corresponding to embolic volumes from 0 to 4.0 mL (in 0.5 mL steps). Each simulation spanned 1.6 s (two cardiac cycles) with a time step of 0.005 s and a convergence criterion of 1 × 10^−3^. A maximum of 100 iterations was allowed per step, and under‐relaxation factors were set to default Fluent values. To eliminate startup transients, only the final 0.8 s were used for data analysis. These waveform segments were concatenated along the time axis to visualize the pressure and flow changes under progressive embolization conditions. While this does not reflect a continuous timeline, it provides intuitive insight into flow‐pressure evolution during embolization.

### 2.3 In vitro experiments

#### 2.3.1 In vitro experimental platform

**FIGURE 4:**
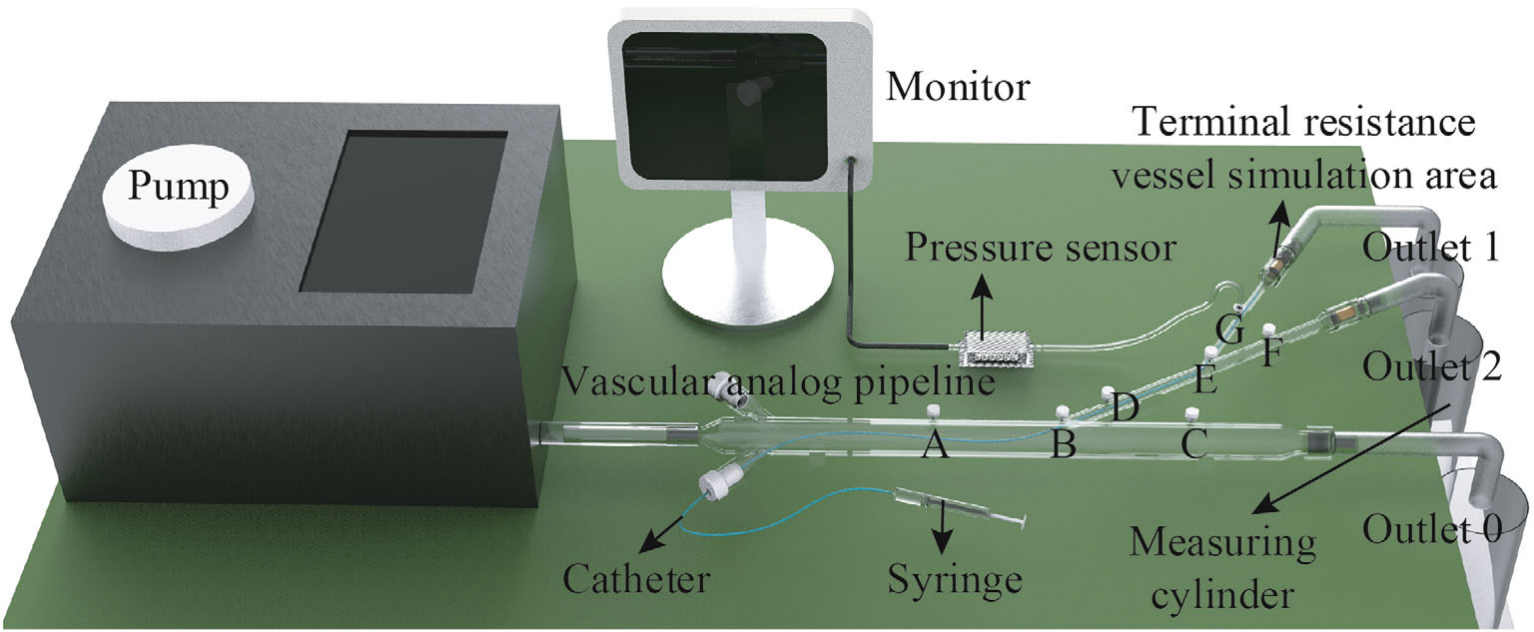
In vitro experiment platform.

The in vitro experimental setup, as illustrated in Figure 4, consists of four key components: a pulsation pump, embolism simulation area, pressure sensor, and a 3D‐printed vascular phantom. The pulsation pump (Ningbo Trando 3D Medical Technology Co., Ltd., China) provides programmable control over pressure and flow settings to mimic physiological arterial waveforms. The vascular simulation pipeline, fabricated from transparent resin using 3D printing, replicates the geometry of the unit vessel model. To facilitate local pressure monitoring, pressure measurement ports were embedded at designated locations along the vascular model, each designed with a standardized interface that allows direct connection to a pressure transducer via a Ruhr connector. This setup enabled real‐time acquisition of systolic, diastolic, and mean arterial pressure (MAP). The pressure transducer (Abbott 42584‐05, ICU Medical, Inc., California, USA) are installed to measure internal pressure.

Embolic particles should enter and be intercepted within the porous media to simulate the embolic process. We propose a structure using three copper sponges with average pore sizes of 0.4, 0.3, and 0.2 mm in the flow direction.^11^ Copper sponges, known for their stability and availability, serve as typical porous media. We used three sponges stacked sequentially with average pore sizes of 0.4, 0.3, and 0.2 mm to create a variable‐path channel for embolic agents. As embolic particles enter the sponge, they are trapped in the final segment due to the decreasing pore size, continuously accumulating to simulate endovascular embolism.

Alternative products resembling clinical embolic agents were selected to reduce costs. The embolic particles are plastic microspheres with 200, 300, and 400 μm diameters (Zhiyi Microsphere Technology Ltd.). Equal masses of these three types of microspheres were combined to make 1 g. Following this, 20 mL of xanthan gum solution (comprising 0.4 g xanthan gum, 6 g salt, and 1 L water) was thoroughly mixed with the microspheres, ensuring they were suspended in the solution. The brine was tinted red to assist in observing the flow of the embolic agent into the pipeline.

#### 2.3.2 In vitro experimental process

The objective of this in vitro experiment was to monitor changes in local arterial pressure following embolic agent injection, and to record the pressure and flow information under different embolic degrees. The purpose was to analyze the feasibility of local pressure as the parameter used to evaluate the embolic endpoint.

A physiologically realistic pulsatile flow was generated using the programmable pump, configured according to a standardized pressure–flow parameter matrix provided by the manufacturer. Once the pulse pump was activated, we measured the volume of liquid collected at each outlet in 15 s using a measuring cylinder and converted it to the flow. A pressure sensor was utilized to measure the pressure at each designated point. Following this, the outlet of the embolic branch was intentionally obstructed to create a fully embolized state. After re‐measuring the local pressure, the flow within the embolic branch was reinstated.

A total of 0.5 mL of embolic agent was injected during each cycle using a syringe through a 4Fr microcatheter, with red dye added to the suspension to visually monitor potential reflux. As embolization progressed and resistance increased, the injection rate was gradually reduced to avoid retrograde flow. After each injection, the catheter was flushed with 20 mL of water to prevent residual microspheres from interfering with the next injection. After each injection, we recorded the mean arterial pressure (MAP) once the waveform stabilized, typically within 5–10 pulsatile cycles.

Finally, when a small number of embolic particles accumulated in front of the embolic branch copper sponge (approximately 4 mL of embolic agent, as confirmed by several experiments), we concluded that the endpoint of embolization had been reached, and the injection was halted.

To ensure the reliability and repeatability of the findings, the entire experimental process was independently repeated three times under identical conditions. For each trial, pressure and flow data were recorded after every injection step. Final results are reported as mean ± standard deviation across all three replicates.

### 2.4 Animal experiments

Pigs are the most widely used animal model in preclinical studies involving microspheres.^24^ They closely resemble humans in terms of anatomical size and structure, physiology, immunology, and genome. In particular, pig kidneys are similar to human kidneys in size, vascular diameter, and arterial length,^25^ making them suitable for this study. If ectopic embolization of microspheres occurs in pig kidneys, anatomical examination can easily detect it.^26^ The overall embolization procedure in pigs closely resembles that in clinical practice. Hence, the pig model can effectively simulate transcatheter arterial embolization.

All animal procedures were approved by the Institutional Animal Care and Use Committee of Fudan University, and conformed to the ethical standards set by the Animal Experiment Committee of Zhongshan Hospital, Fudan University. Three pigs were enrolled in this study, designated as Pigs 1, 2, and 3, with body weights of 62.8, 58, and 60 kg, respectively. Pigs 1 and 2 were operated on at Gateway Medical Innovation Center (Shanghai, China), and Pig 3 at Shanghai Xinova Medical Research Co., Ltd.

**FIGURE 5:**
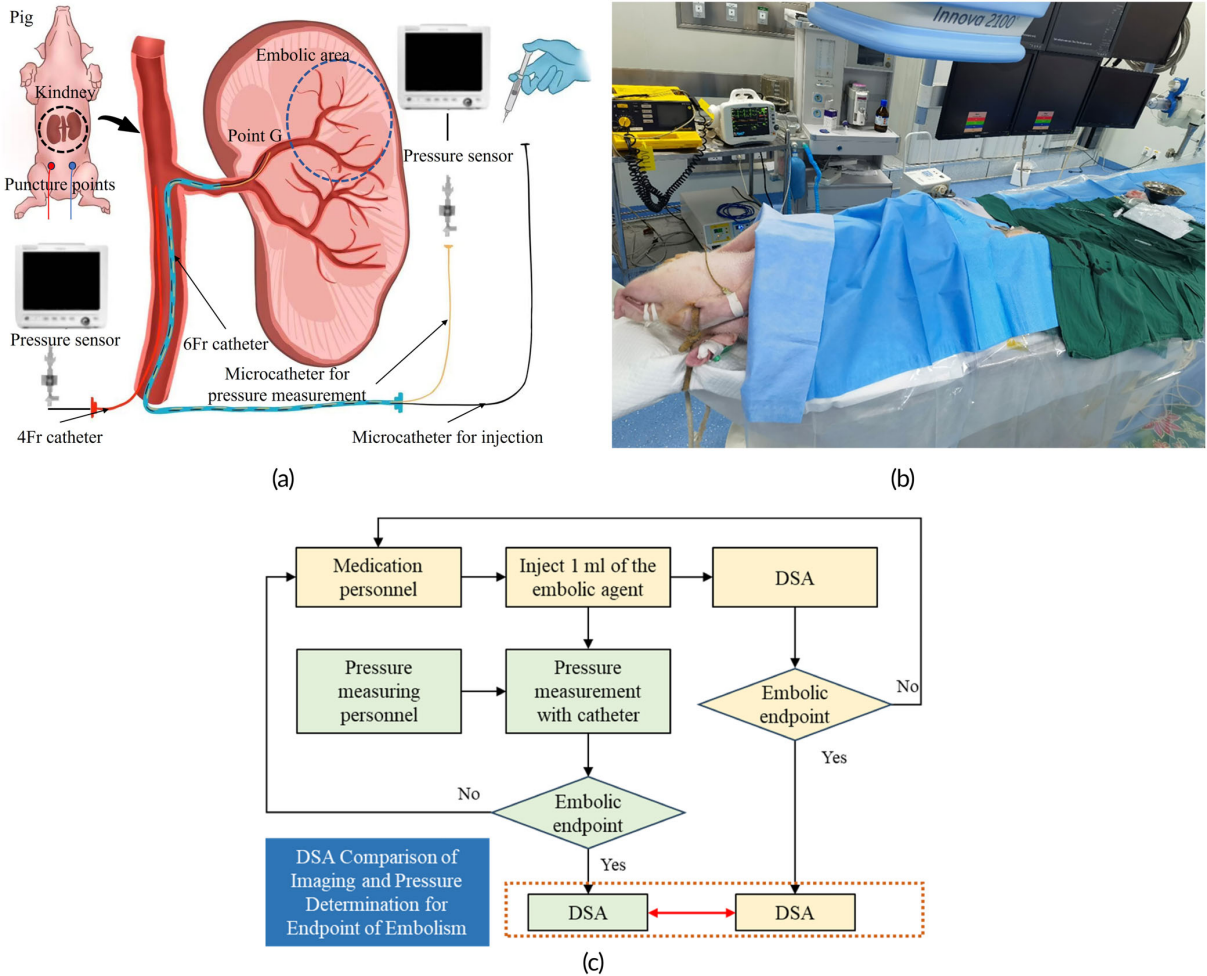
Programs for animal experiments: (a) Schematic of the experimental setup. (b) Image of the surgical site. (c) Implementation strategy of the two endpoints of embolization discrimination methods in the experiment.

The experimental setup is shown in Figure 5a. Two distinct types of pressure measurements were obtained to support embolization assessment: baseline pressure and final pressure. The baseline pressure was defined as the mean arterial pressure (MAP) at Point B, located in the abdominal aorta at the level of the renal artery orifice, recorded over a stable period of 5–10 cardiac cycles prior to the first embolic injection. This value served as the normalization reference in Equation (6), helping eliminate the influence of systemic fluctuations across animals. The final pressure was defined as the local arterial pressure at Point G1, measured under complete downstream occlusion. To acquire this, a balloon catheter was temporarily inflated to block the embolized vessel, simulating the terminal resistance condition. The pressure measured at this point reflects the maximum achievable upstream pressure when perfusion is fully obstructed. These two reference values represent different physiological contexts: baseline pressure captures the systemic hemodynamic environment before embolization, while final pressure provides an upper boundary for local pressure buildup during embolization. Both were essential for defining and validating the pressure‐based embolization index proposed in this study.

Under 0.014 guidewire guidance, a 3Fr microcatheter was introduced via the 6Fr into the secondary branch artery at the upper pole of the left kidney. The leading edge of the microcatheter, along with the anatomical structure and blood flow status of the target vessel, was determined using DSA fluoroscopy. Once the embolic microcatheter was correctly positioned, a mixture of embolic microspheres and a contrast agent (1 g of microspheres, 7 mL of normal saline, and enough contrast agent to make up 20 mL, thoroughly shaken) was injected into the target artery through the catheter at an average rate of less than 0.5 mL/min. Pressure measurements were performed via a second dedicated 3Fr microcatheter connected to a clinical‐grade pressure transducer. The pressure‐sensing catheter was positioned proximally to the embolization zone (closer to the aorta) to capture the upstream arterial response to embolization. The mean pressure was recorded after following each 1 mL injection of the embolic agent 1 min. Figure 5b depicts the animal experiment site. Throughout the procedure, MAP remained below 100 mmHg and systolic pressure remained below 130 mmHg, well within the normal physiological range for anesthetized pigs reported in the literature.^27^

As shown in Figure 5c, the doctor responsible for injecting embolic agents relied solely on the images to determine the endpoint of the embolism. Simultaneously, the measuring personnel were blinded to the image and determined the embolization endpoint based purely on pressure trends. Finally, the feasibility of determining the endpoint of embolization based on the local arterial pressure was validated via image comparison. Considering the safety factors, the endpoint of embolization was reached when the embolic degree assessed by the local arterial pressure was above 95%. DSA images at the first instance of more than 95% embolization were utilized as the final images for the pressure assessment.

## 3 RESULTS

### 3.1 Simulation

**FIGURE 6:**
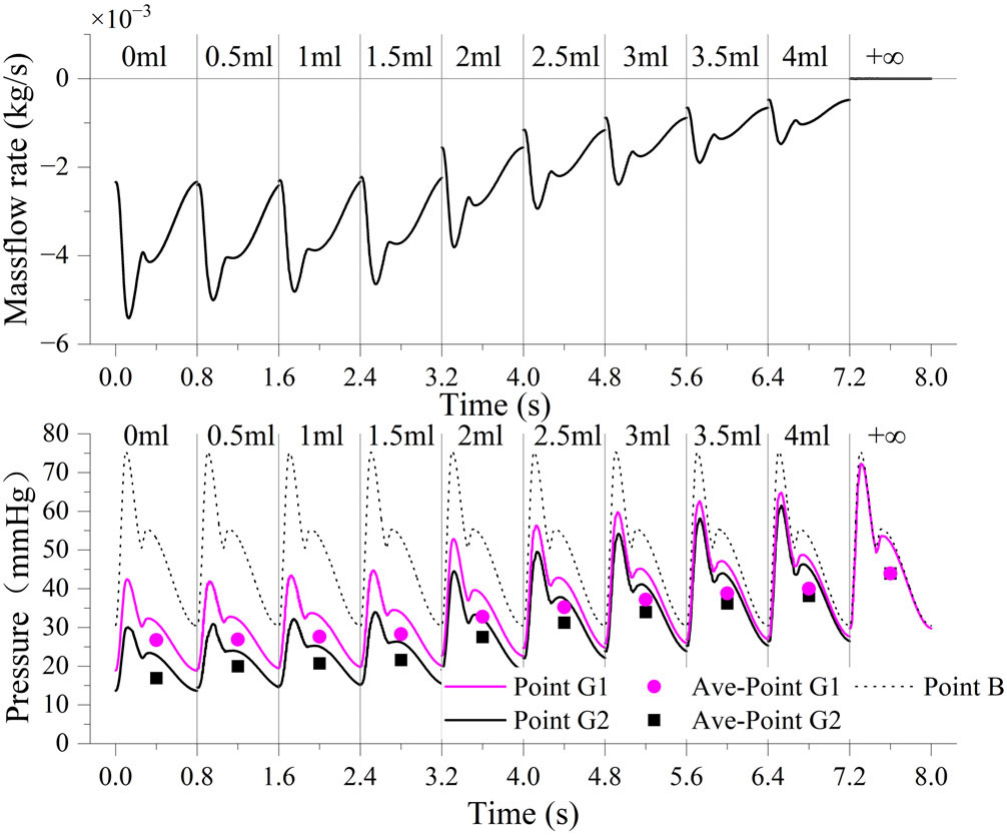
Simulation results for different embolization states. The x‐axis is composed of concatenated 0.8‐s waveform segments, each obtained from a separate simulation corresponding to a specific embolization volume. Although the waveform appears continuous, the time axis does not represent a real‐time timeline, but rather serves to illustrate stage‐wise hemodynamic changes in pressure and flow.

Figure 6 illustrates the simulated pressure waveforms at Point B (abdominal aorta) and Points G1 and G2 (distal arterial branches), as well as the mass flow rate across Plane 1, under various stages of embolization. The waveform data between 7.2 and 8.0 s represent the fully embolized state, simulated by sealing Outlet 1 to indicate complete occlusion.

At the early stages of embolization (0–1.5 mL), the local arterial pressures at Points G1 and G2 remained relatively stable, indicating minimal resistance to flow. As embolic agents progressively accumulated, systolic pressure increased substantially—peaking at 29.88 mmHg (a 70.49% increase) at Point G2—and the waveform shape gradually began to resemble that of the abdominal aorta in terms of amplitude and peak timing. This reflects a transition toward upstream dominance in pressure transmission, likely resulting from elevated downstream resistance and reduced microvascular compliance.

The mean arterial pressure (MAP) was calculated as:

(5) $$\mathrm{MAP}=\text{diastolic pressure}+\left(\text{systolic pressure}-\text{diastolic pressure}\right)/3.$$

During embolization, the mean arterial pressure (MAP) increased by 17.21 mmHg (a 64.36% rise) at Point G1, and by 24.53 mmHg (a 126.38% rise) at Point G2. In contrast, the pressure waveform at Point B exhibited negligible variation throughout the embolization process, indicating that the hemodynamic changes were localized rather than systemic.

Plane 1, as illustrated in Figure 2, is positioned immediately proximal to the microcatheter tip. A negative mass flow rate across this plane indicates forward perfusion from the proximal to distal segment. As the embolic dose increased, this flow rate progressively declined, ultimately reaching zero—signifying complete flow cessation at the embolization endpoint.

These findings reveal a physiologically consistent response: as embolic material accumulates in the target branch, downstream vascular resistance increases, resulting in a stepwise elevation in local arterial pressure and a corresponding reduction in flow. This trend culminates in complete occlusion, aligning with the expected hemodynamic consequences of successful vascular embolization.

### 3.2 Embolism endpoint assessment methodology

**FIGURE 7:**
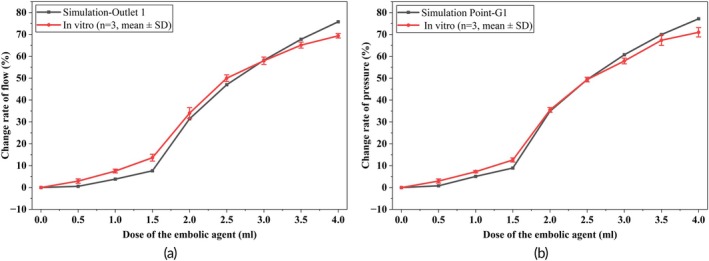
Result of embolization assessment with the flow and local pressure: (a) Assessment of the embolism based on the flow and (b) assessment of the embolism based on the local arterial pressure. Data from in vitro experiments are presented as mean ± SD.

Figure 7 presents the results of embolization assessment using based on both blood flow rate (Figure 7a) and local arterial pressure (Figure 7b). These results were derived from simulation and in vitro experiments, with the latter based on three repeated trials. The in vitro data are shown as mean ± standard deviation, capturing inter‐trial variability and enabling direct comparison with simulated outcomes.

Traditionally, the endpoint of embolization is determined by visual assessment of residual flow in the target vessel using DSA imaging. To explore a more quantitative and objective alternative, we collected local arterial pressure and branch flow data from both simulations and in vitro experiments, and examined their relationship with cumulative embolic dose. While blood flow is a direct indicator of embolic degree, its real‐time intraoperative measurement remains difficult due to sensor limitations and vessel scale constraints. In practice, physicians also infer flow reduction during DSA‐based assessment.

Our results reveal a strong correlation between mean local arterial pressure and corresponding changes in blood flow across all embolization stages. This relationship was consistently observed in both simulations and experiments, as shown in Figure 7. When comparing embolization degrees estimated from flow reduction versus pressure elevation, the average deviation was only 1.65% in simulations and 3.09% in vitro experiments. These findings demonstrate the feasibility of replacing subjective image‐based evaluations with a pressure‐driven approach, offering an objective and real‐time method for embolization endpoint determination.

Considering that systolic and diastolic pressure fluctuate dynamically during embolization, we selected mean arterial pressure (MAP) as a stable indicator. Clinically, MAP is already used in diagnostic indices such as fractional flow reserve (FFR) for coronary stenosis assessment.^28^ Inspired by the FFR concept, we developed a pressure‐based method to quantitatively evaluate the embolization degree. To eliminate confounding influences from upstream pressure fluctuations (e.g., baseline variability or systemic compensation), we introduced a normalization approach using the abdominal aortic pressure at Point B as a reference. This led to the following embolization degree formula:

(6) $$\begin{array}{c} F\left(t\right)=\frac{P_{G-\mathrm{REA}-\mathrm{AVE}}^{\prime }\left(t\right)-P_{G-\mathrm{AVE}}\left(t=0\right)}{P_{G-\mathrm{FIN}-\mathrm{AVE}}-P_{G-\mathrm{AVE}}\left(t=0\right)}\times 100\% \end{array},$$

$F\left(t\right)$ denotes the degree of embolization over time, $P_{G-\mathrm{REA}-\mathrm{AVE}}\left(t\right)$ represents the average pressure of Point G at time *t*, $P_{G-\mathrm{AVE}}\left(t=0\right)$ refers to the pressure at Point G before the procedure, and $P_{G-\mathrm{FIN}-\mathrm{AVE}}$ denotes the final pressure at the end of embolization. In clinical practice, the real‐time mean pressure can be obtained using a pressure transducer, pressure wire, or catheter, and the final pressure acquisition at the end of the embolization requires further discussion. $P_{G-\mathrm{REA}-\mathrm{AVE}}^{\prime }\left(t\right)$ represents the converted real‐time average pressure of Point G.

(7) $$\begin{array}{c} P_{G-\mathrm{REA}-\mathrm{AVE}}^{\prime }\left(t\right)=P_{G-\mathrm{REA}-\mathrm{AVE}}\left(t\right)-\\ \left[P_{B-\mathrm{REA}-\mathrm{AVE}}\left(t\right)-P_{B-\mathrm{REA}-\mathrm{AVE}}\left(t=0\right)\right]\times a, \end{array}$$

where $P_{B-\mathrm{REA}-\mathrm{AVE}}\left(t\right)$ and $P_{B-\mathrm{REA}-\mathrm{AVE}}\left(t=0\right)$ denote the mean pressure at Point B (abdominal aorta or basal blood pressure). Coefficient a represents the pressure transfer coefficient, the loss of pressure fluctuation from Point B to Point G, which is mainly specified by the experiment, clinical data, or experience.

### 3.3 Animal experiments

Renal artery embolization was performed in three pigs (designated as Pig 1, Pig 2, and Pig 3). The embolic agent was injected in 1 mL increments per stage. A total of 8 mL of embolic agent was administered in Pig 1 and Pig 2, and 7 mL in Pig 3. During each embolization stage, both abdominal aortic pressure and renal artery pressure were continuously monitored.

**FIGURE 8:**
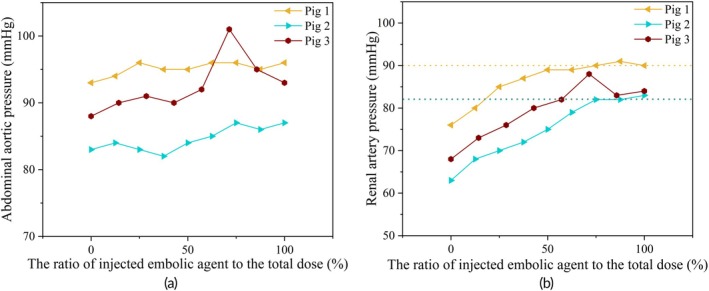
Abdominal aorta and local artery mean pressure in animal experiments: (a) Changes in abdominal aortic pressure and (b) changes in renal artery pressure.

Figure 8a shows the changes in abdominal aortic pressure during the embolization process. All three pigs exhibited a gradual increase in aortic pressure during the injection phases. However, Pig 3 displayed a transient spike at approximately 75% of the total dose, which was likely caused by a compensatory cardiovascular response or drug‐induced stimulation. This fluctuation underscores the potential risk of misjudging the embolization endpoint if solely relying on the instantaneous value of the “final pressure.”

Figure 8b presents the corresponding renal artery pressure curves for each pig. The dashed lines indicate the pressure measured under temporary balloon occlusion, which served as a reference for complete embolization. In all animals, renal artery pressure increased progressively with each embolic injection. However, in the later stages (beyond approximately 85% of the total dose), the pressure curves began to plateau, suggesting that downstream perfusion had been substantially diminished, and that additional embolic agent had minimal further impact on the hemodynamic status.

**FIGURE 9:**
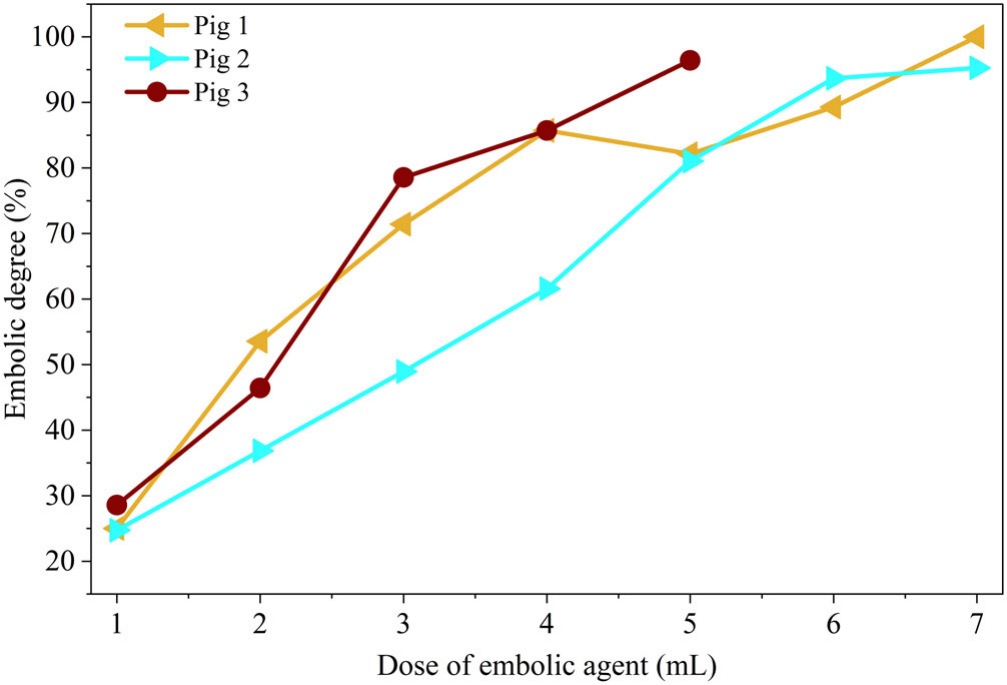
Pressure‐derived embolization degree in three pigs following each 1 mL embolic agent injection.

The degree of embolization after each injection was quantitatively assessed using the pressure‐based index described in Equation (6). In the animal experiments, the baseline pressure was defined as the mean arterial pressure (MAP) recorded after waveform stabilization over 5–10 cardiac cycles, prior to the first embolic injection. This approach minimized the effects of transient hemodynamic fluctuations or drug‐induced variations, ensuring a stable reference for subsequent comparisons. Although the baseline MAP values varied among the three pigs (Figure 8b), each animal served as its own control, and pressure changes were analyzed relative to its individual baseline. According to this index, Pig 1 and Pig 2 reached the embolization endpoint after the seventh injection, while Pig 3 reached it after the fifth. Figure 9 presents the stepwise assessment of embolization degree following each 1 mL embolic agent injection, as calculated from the normalized local arterial pressure using Equation (6). The pressure‐derived embolization index indicated that the endpoint had already been reached after the seventh injection in Pigs 1 and 2, and after the fifth injection in Pig 3.

**FIGURE 10:**
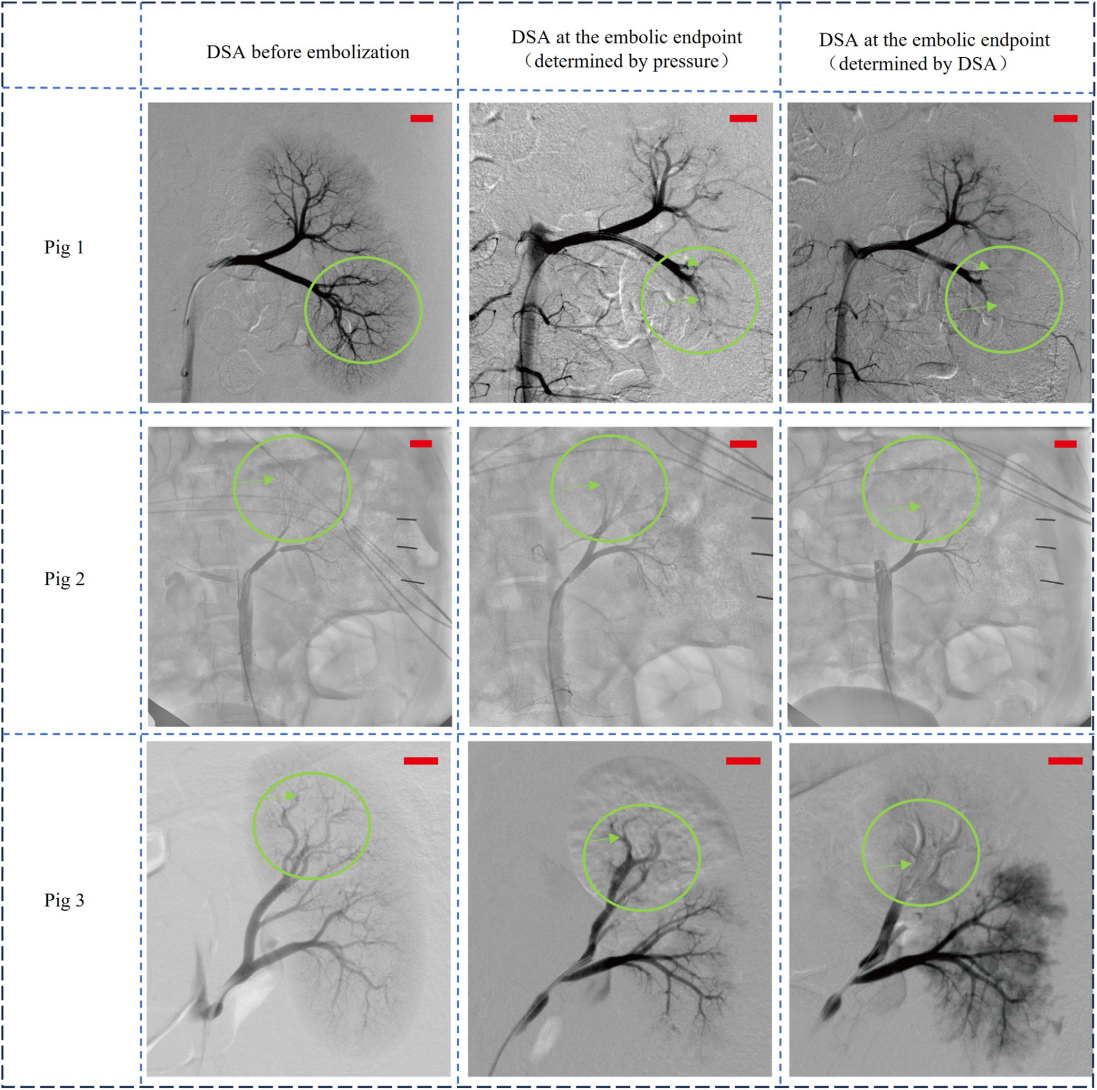
Renal vascular DSA imaging in the pigs before and after embolization (Scale bar: 10 mm).

To compare the effectiveness of the pressure‐based and conventional DSA‐based endpoint assessments, both methods were applied in parallel during animal experiments. At each embolization stage, 1 mL of embolic agent was injected, and DSA images were captured at the decision points determined by each method (Figure 10).

In all three experiments, the baseline images showed well‐perfused renal arterial trees with full distal branch visualization. At the pressure‐based endpoint, over 90% of distal vessel opacification had disappeared, indicating a substantial reduction in perfusion and suggesting adequate embolization. In contrast, the DSA‐determined endpoint occurred after additional embolic agent injection, but with only minimal change in distal visibility. This indicates that pressure monitoring can identify an earlier yet physiologically sufficient embolization endpoint.

**FIGURE 11:**
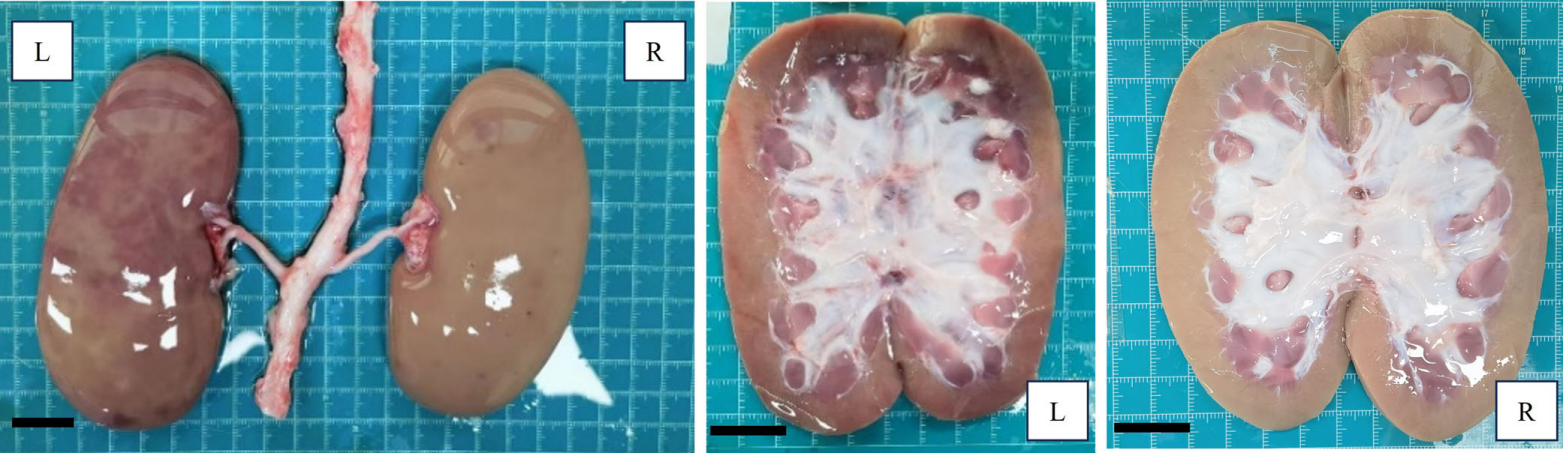
Gross anatomical images of excised pig kidneys after embolization (Scale bar: 20 mm).

Importantly, no reflux of embolic agent was observed at the pressure‐guided endpoints, and embolic coverage appeared comparable between the two methods. These findings suggest that pressure‐guided assessment may help reduce overtreatment and embolic burden without compromising embolization efficacy. Figure 11 presents the gross anatomy of the kidney from Pig 3, clearly showing a boundary between embolized and non‐embolized regions. Similar vascular congestion patterns were observed in the other animals as well, confirming the success of embolization.

After determining the embolization endpoint using two different approaches—pressure‐based and DSA‐based—the corresponding DSA images were anonymized and submitted for independent evaluation. Five board‐certified interventional radiologists (with ≥5 years of clinical experience) from Zhongshan Hospital, Fudan University, were invited to assess the embolization outcomes using a standardized scoring rubric (Table 2). Their evaluation results are summarized in Table 3.

**TABLE 2:** Criteria for evaluating the degree of embolization.

Evaluation grade	Evaluation criterion
A	Complete embolization. No contrast staining is visible in any of the target arteries.
B	Incomplete embolization. Contrast staining in the target vessels is reduced, but significant perfusion remains. Non‐target artery flow remains unaffected. There may be substantial reduction in smaller branches (Level 2 and below), but not all target vessels are fully embolized.
C	Over‐embolization. Contrast staining is reduced or absent in non‐target arteries, suggesting unintended embolization beyond the target region.

**TABLE 3:** Evaluation results for the degree of embolization.

Doctor	Pig 1		Pig 2		Pig 3	
	Pressure	DSA	Pressure	DSA	Pressure	DSA
1	A	A	B	A	A	A
2	A	A	B	A	A	C
3	A	A	A	A	A	A
4	B	A	A	A	B	C
5	A	A	A	A	A	A

While physician expertise inevitably influences visual judgment, the radiologists emphasized that embolization assessment should always be interpreted in the context of procedural intent and vascular anatomy. Despite minor variations in individual scores, the majority of clinicians agreed that the two sets of DSA images—those acquired at the pressure‐based endpoint and those captured at the DSA‐based endpoint—demonstrated comparable embolization efficacy.

These findings highlight the inherent subjectivity of image‐based assessment and reinforce the need for quantitative, objective criteria to guide embolization endpoint determination in clinical practice.

## 4 DISCUSSION

Effective reconstruction of the embolization process is essential for accurate analyzing hemodynamic parameters. A porous media model describing the embolic process offers an innovative method to avoid physically modeling complex capillary networks. Fluid simulations and in vitro experiments modeled the end vessel network as a porous medium equivalent. The simulation and experimental outcomes confirmed a close correlation between local arterial pressure and the degree of embolism. Inspired by the clinical use of mean arterial pressure (MAP) in the diagnosis of coronary artery stenosis, this study developed a novel pressure‐based method to evaluate embolization endpoints. The efficacy of this method was further supported by animal experiments. The proposed approach demonstrates potential for clinical application, offering a quantifiable, real‐time tool to assist interventional radiologists in objectively determining embolization endpoints.

The hemodynamic behavior observed during progressive embolization in the simulation reveals key nonlinear and saturable characteristics. As embolic agent volume increased, local arterial pressure exhibited an initial rapid rise followed by a plateau, while mass flow rate gradually decreased and approached zero. This trend reflects the diminishing marginal impact of embolic agent as downstream resistance saturates, a hallmark of pressure‐flow coupling in confined vascular territories.

To enable stage‐wise comparison, the simulated waveform segments—each derived from independent transient simulations—were aligned along a virtual timeline. Although discontinuities are present at the segment boundaries, they do not affect the interpretation of embolization dynamics and serve to highlight phase transitions in pressure and flow. This composite visualization facilitated the identification of three distinct embolization phases: a low‐resistance phase with minimal hemodynamic change, a transitional phase with rapidly rising pressure, and a plateau phase indicating near‐complete occlusion.

In addition to amplitude changes, waveform morphology also evolved significantly. The progressive attenuation and delay of the dicrotic notch suggest a loss of flow pulsatility and increasing impedance mismatch within the vascular network. These observations are consistent with pressure wave reflections caused by abrupt changes in geometry or compliance. Although wall elasticity and wave transmission effects were not explicitly modeled in this study, the evolving waveform features indirectly point to their physiological relevance. Future studies incorporating fluid–structure interaction or transmission line models may offer deeper insights into the biophysical basis of these phenomena.

In the animal experiments, a transient and irregular spike in aortic pressure was observed in Pig 3, as shown in Figure 8a. his fluctuation likely reflects a compensatory physiological response triggered by drug stimulation or injection stress. A corresponding pressure peak was also recorded in the local arterial branch, which, if interpreted in isolation, could lead to misjudging the embolization endpoint when using final pressure alone. To address such variability, the proposed pressure‐based index in Equation (6) incorporates normalization against baseline pressure at the abdominal aorta, thereby reducing sensitivity to systemic fluctuations.

As demonstrated in Figure 10, the embolization degree estimated using local pressure was generally consistent with the DSA‐based physician judgment, and even indicated an earlier endpoint in Pig 3. Beyond this point, additional embolic agents primarily accumulated in secondary branches near the catheter tip, which are often not clinically required to be fully occluded. Over‐embolization in these regions could increase the risk of reflux and downstream complications, suggesting that pressure‐guided early termination may improve procedural safety while reducing material use. This has particular significance for expensive therapeutic agents like 90Y microspheres.

These findings highlight the potential of pressure‐derived embolization assessment as a control parameter for automation. Future work will focus on integrating the local arterial pressure quantification method into robotic systems, potentially enabling real‐time feedback and adjustment during interventional procedures. Additionally, the method's applicability across different types of embolic agents and varying clinical conditions will be explored. Automatic drug injection devices are primarily utilized for controlling the depth of anesthesia during clinical procedures or for clinical regulation of recovery of a single physiological index.^29^, ^30^, ^31^ In interventional procedures, automated drug injections are mainly used for contrast agents. Due to the high operational risks, the control system must meet stringent real‐time and safety requirements. Conventional timed or quantitative injection modes do not meet the criteria for embolic agent injection. Embolic agent injection robot with physiological information feedback ability is possible to improve the success rate and safety of interventional embolization, and reduce the radiation exposure of doctors and patients. Utilizing local blood pressure as a control parameter could markedly improve the safety and feasibility of automatic embolic injection. This method would lessen the dependency on imaging information for identifying endpoint of embolization. However, the discrepancy between real‐time pressure during injection and post‐injection pressure must be addressed in future system designs.

Although DSA imaging remains the standard for embolization endpoint judgment, it is highly operator‐dependent. The proposed pressure‐based method offers a quantitative, reproducible supplement to visual interpretation, which may support greater clinical standardization. Further clinical trials are needed to validate this approach across various embolization types and patient conditions.

## 5 CONCLUSIONS

This study presents a novel and quantitative approach for determining the embolization endpoint, designed to enhance accuracy, repeatability, and safety in interventional embolotherapy. By representing the terminal microvasculature as a porous medium, the method enables efficient simulation of hemodynamic changes during progressive embolization. This modeling strategy was integrated with in vitro experiments and further validated through in vivo renal artery embolization in pigs, confirming the reliability and translational potential of the proposed method.

Unlike conventional image‐based assessments that are subjective and operator‐dependent, the pressure‐based method offers a standardized, real‐time, and sensor‐driven evaluation framework. Its quantitative nature allows for precise endpoint detection, thereby reducing the risk of overtreatment and embolic reflux. Clinically, this technique holds promise for improving the consistency of embolization outcomes, maximizing the preservation of normal tissue, and minimizing radiation exposure.

Moreover, this approach lays the groundwork for future intelligent and automated embolic delivery systems, where real‐time physiological feedback—such as local arterial pressure—can guide robotic injection protocols with greater precision and autonomy.

## AUTHOR CONTRIBUTIONS

**Dongcheng Ren**: Methodology; writing—original draft; validation, data curation, funding acquisition. **Xingyuan Li**: Writing—review and editing; investigation. **Shijie Guo**: Conceptualization; funding acquisition; supervision. **Yuchi Tian**: Writing—review and editing; formal analysis. **Baolei Guo**: Validation; funding acquisition; writing—review and editing. **Bo Zhou**: Conceptualization; validation; funding acquisition; project administration; supervision.

## CONFLICT OF INTEREST STATEMENT

The authors declare no conflicts of interest.

## Data Availability

The data that support the findings of this study are available from the corresponding author upon reasonable request.
